# Osteoarthritic changes in the knees of recently retired male professional footballers: a pilot study

**DOI:** 10.17159/2078-516X/2022/v34i1a12816

**Published:** 2022-01-01

**Authors:** S Carmody, H Aoki, O Kilic, M Maas, A Massey, GM Kerkhoffs, V Gouttebarge

**Affiliations:** 1 Amsterdam UMC location University of Amsterdam, Department of Orthopedic Surgery and Sports Medicine, Meibergdreef 9, Amsterdam, The Netherlands; 2 Medical Department, Chelsea Football Club, London, United Kingdom; 3 St. Marianna University School of Medicine, Kawasaki, Japan; 4 Yokohama City Sports Medical Center, Yokohama, Japan; 5 Amsterdam Collaboration on Health & Safety in Sports (ACHSS), IOC Research Center, Amsterdam, The Netherlands; 6 Amsterdam UMC location University of Amsterdam, Department of Musculoskeletal Radiology, Meibergdreef 9, Amsterdam, The Netherlands; 7 Academic Center for Evidence based Sports medicine (ACES), Amsterdam, The Netherlands; 8 Medical Department, Fédération Internationale de Football Association (FIFA), Zurich, Switzerland; 9 Amsterdam Movement Sciences, Aging & Vitality, Musculoskeletal Health, Sports, Amsterdam, The Netherlands; 10 Section Sports Medicine, University of Pretoria, Pretoria, South Africa; 11 Football Players Worldwide (FIFPRO), Hoofddorp, The Netherlands

**Keywords:** osteoarthritis, knee injuries, professional footballers, retirement

## Abstract

**Background:**

Knee osteoarthritis (OA) is common amongst retired male professional footballers. There is limited understanding with respect to the interplay between imaging findings, clinical presentation and patient-reported outcome measures (PROMs) in retired professional footballers with knee OA.

**Objectives:**

This pilot study aimed to evaluate the extent of radiological and clinical knee OA in a cohort of retired male professional footballers, and to explore the relationship between these findings and knee-related PROMs.

**Methods:**

Fifteen retired male professional footballers underwent knee radiographs and were surveyed on their history of clinical OA, severe knee injury and previous knee surgery. The Knee Injury and Osteoarthritis Outcome Score Physical Function Short Form (KOOS-PS) and the Patient-Reported Outcomes Measurement Information System Global Health (PROMIS-GH) were used to assess health outcomes, such as level of function and pain.

**Results:**

Radiological knee OA was diagnosed in six out of 15 participants. Seven of the participants had a clinical diagnosis of knee OA. Evidence of clinical and radiological OA was present amongst four participants. Radiological knee OA and clinical OA was significantly associated with a history of severe knee injury and previous knee surgery. Low correlations (ρ<-0.40) were found between knee OA severity and knee-related PROMs. Moderate correlation (ρ=-0.65) was found between clinical knee OA and KOOS-SP.

**Conclusion:**

Clinical knee OA correlates with PROMs amongst retired professional footballers but radiological OA does not. Further studies are required to understand the relationship between imaging findings, clinical presentation and PROMs amongst retired professional footballers with knee OA.

Knee injuries are common amongst male professional footballers (soccer players).^[1]^ The management of these injuries may occasionally require surgical intervention, and this, along with the impact of repetitive joint loading, compounds the risk of complications, such as early-onset knee osteoarthritis (OA) for footballers in later life.^[2]^ Knee OA is prevalent amongst retired professional footballers, with studies reporting a high prevalence ^[2][3][4]^, although it is not currently considered an occupational disease.^[7]^ It is a disabling condition manifesting in pain, swelling, stiffness and loss of range of motion. Knee OA and its sequelae place a significant health burden on individuals and healthcare services worldwide, with affected individuals often unable to work, dependent on medication to complete activities of daily living (ADLs), and in many cases requiring surgical intervention (e.g. total knee replacement, TKR).^[5]^

The diagnosis of knee OA, based on suggestive clinical features (e.g. activity-related joint pain, functional impairment), is often supported by the presence of joint space narrowing on radiographic examination.^[6]^ Previous studies have estimated the prevalence of knee OA in retired footballers to be as high as 80%,^[4]^ with retired footballers three times more likely to report a diagnosis of knee OA than those in an age-matched general population.^[2]^ The interpretation of this high prevalence is limited by the fact that the definition of knee OA varies across epidemiological studies, with radiological (e.g. x-ray) knee OA, clinical knee OA (e.g. physical signs and symptoms), self-reported knee OA, all being considered in addition to those on the waiting list for (or having received) knee arthroplasty for knee OA. While OA is thought to be associated with reduced quality of life and mental health symptoms amongst retired athletes,^[8]^ there is limited evidence examining the relationship between the extent of radiological knee OA (e.g. joint space narrowing) and Patient Reported Outcome Measures (PROMs) related to pain/impairments and quality of life.

PROMs are used in OA to assess domains, such as pain, functional status and structural damage. Established PROMs, such as the Western Ontario McMaster Universities’ Osteoarthritis Index (WOMAC) and the Knee Injury and Osteoarthritis Outcome Score (KOOS), are the most common measures of functional status that have been used for knee OA.^[9][10]^ The seven-item Knee Injury and Osteoarthritis Outcome Score Physical Function Shortform (KOOS-PS) is an abbreviated PROM and has been shown to be valid and reliable for assessing the impact of OA on patient function.^[11]^ Understanding the relationship between the extent of OA, quality of life, and function amongst retired professional footballers may provide insight into potential interventions to improve outcomes in this cohort.

Therefore, the primary objective of this pilot study was to evaluate the extent of radiological and clinical knee OA in a cohort of retired male professional footballers, and to explore the relationship between these findings and knee-related PROMs.

## Methods

### Study design

An observational study based on a cross-sectional design was conducted, using the Strengthening the Reporting of Observational Studies in Epidemiology (STROBE) statement in order to ensure a high quality of reporting.^[12]^ Ethical approval for the study was provided by the Ethical Committee of the Yokohama City Sports Medical Center (17.003; Yokohama, Japan) and the Medical Ethics Review Committee of the Academic Medical Center, (W16_366#16.431) Amsterdam, The Netherlands). The study was conducted in accordance with the principles set out in the Declaration of Helsinki (2013).^[13]^ Players participated voluntarily in the study and did not receive any financial remuneration for their participation.

### Participants

A sample of 15 recently retired professional footballers (six Finnish, four Norwegians, three Dutch, two Americans) was recruited by Football Players Worldwide (FIFPRO), the international union for professional footballers. The inclusion criteria were: (1) retired professional football player, (2) aged up to 50 years, (3) male sex and (4) ability to understand text written in English.

In our study, a retired male professional footballer was defined as an individual who was remunerated for devoting several hours in all/most days (exceeding the time allocated to other types of professional or leisure activities) to playing football, and in which they competed in the highest or second highest national league.

The following exclusion criterion was defined: those with a confirmed diagnosis of other forms of arthritis or systemic medical conditions with a predilection for joint manifestations.

### Radiological and clinical knee osteoarthritis

Two-sided weight-bearing knee radiographs were performed (Rosenberg view, standing anteroposterior view, standing lateral view) according to a standardised protocol. Knee joint space narrowing was assessed by an experienced radiologist (MM) and classified according to Kellgren-Lawrence criteria (from Grade 0 to Grade 4), with OA also being deemed as present at Grade 3 or Grade 4.^[14][15]^ Participants were invited to disclose any confirmed history of clinical knee OA. Clinical knee OA was defined according to the NICE criteria (adapted for age) as damage of the knee joint’s cartilage leading to activity-related joint pain and either no morning joint-related stiffness or morning stiffness that lasts no longer than 30 min.^[16]^

### Knee-related patient-reported outcome measures

The history of severe knee injury and subsequent surgery during a career as a professional footballer was examined by means of a single question; ‘How many severe knee injuries have you had during your career as a professional footballer?’ In our study, a severe knee injury was defined as; ‘an injury that involved the knee joint, occurred during team activities (training or match), and led to either training or match absence for more than 28 days’.^[17]^ The validated Knee Injury and Osteoarthritis Outcome Score Physical Function Short Form (KOOS-PS) was used to assess the level of knee function.^[11]^ A total score ranging from 0 to 100 was calculated, where 0 represented complete knee disability and 100 indicated impeccable knee function.^[18]^ Reference values for physical knee function are available for standard adult populations, young athletic populations, and amongst amateur footballers.^[18][19][20]^ The Patient-Reported Outcomes Measurement Information System Global Health (PROMIS-GH) was used to assess multiple domains related to health-related quality of life, such as physical health, levels of function, pain, social activities and fatigue, for this study.^[21]^ Based on 10 items, each measured on a five-point scale (from one to five), the Global Physical Health and Global Mental Health scores were calculated. These subscale scores ranged from 0 to 100, with a higher score indicating a better quality of life and a mean score of 50 indicating the norm for the general population.

### Procedures

An electronic anonymous questionnaire (see supplementary material) available in English was compiled (LimeSurvey Professional), including for all PROMs, as well as the following descriptive variables: age, height (cm), weight (kg), duration of professional football career, level of play, level of education, duration and nature of retirement, and current employment status. Information about the study was emailed to potential participants by FIFPRO, with the process hidden from the principal researcher for privacy reasons and to minimise the risk of researcher bias. If interested in the study, participants gave their informed consent and were asked to visit a clinic of their choice for the aforementioned standardised radiological assessment. Participants were concurrently asked to complete the electronic questionnaire. The responses to the questionnaires were coded and made anonymous for reasons of privacy and confidentiality. Once completed, all data collected were saved automatically on a secure electronic server that only the principal researcher could access.

### Statistical analysis

The statistical software IBM SPSS 26.0 for Apple Mac was used for data analysis. Descriptive analyses (mean, standard deviation, frequency and range) were performed for all variables included in the study. Correlation between radiological knee OA and clinical knee OA with knee-related PROMs was explored with Spearman’s rank correlation coefficient (ρ).^[22]^

## Results

The respective mean age, height and weight of the participants was 39 years (SD=4), 183 cm (SD=5) and 82 kg (SD=7). Participants must have played professional football for 13 years on average (87% at the highest club level in their country) and been retired for five years. Two (13%) participants were forced to retire through injury. All the characteristics of the participants are presented in Table 1.

**Fig. 1:**
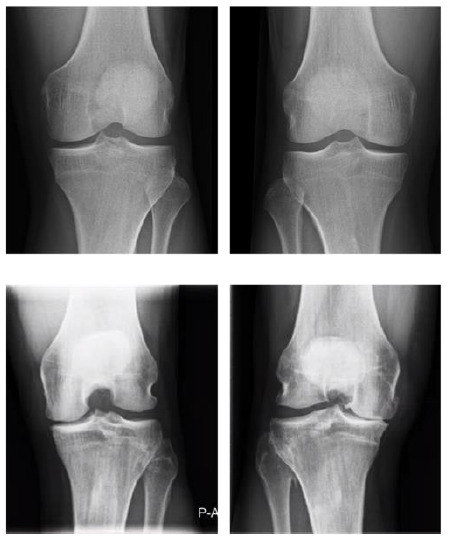
Knee radiographs (Rosenberg view) of two retired professional footballers (top two radiographs: Grade 0; bottom two radiographs: Grade 3–4). The bottom two radiographs demonstrate radiological features of OA including loss of joint space and osteophytes.

The main findings of the study are presented in Table 2. Radiological knee OA was diagnosed in six participants (40%; two bilateral and four unilateral). Seven (47%) of the participants had a clinical diagnosis of knee OA. Four (27%) of the participants had evidence of both clinical and radiographic knee OA. Figure 1 displays the radiographs (Rosenberg view) of two of the participants, one without radiological OA (Grade 0) (top two radiographs) and one with bilateral (Grade 3 and Grade 4) radiological OA (bottom two radiographs). A Chi-square test indicated that radiological knee OA was significantly associated with severe knee injury (χ2 = 3.64; df = 1; p < 0.10) and knee surgery (χ2 = 3.62; df = 1; p < 0.10). AChi-square test indicated that clinical knee OA was significantly associated with both severe knee injury (χ2 = 4.77; df = 1; p < 0.10) and knee surgery (χ2 = 5.53; df = 1; p < 0.10). The mean score on the KOOS-SP was 88. The Global Physical Health and Global Mental Health mean scores were 55 and 54, respectively. Low correlations (ρ<−0.40; p>0.10) were found between radiological knee OA severity and knee-related PROMs. Moderate correlation (ρ=−0.65; p<0.01) was found between clinical knee OA and KOOS-SP.

## Discussion

This pilot study investigated the presence of knee OA amongst retired male professional footballers, and explored the relationship between these findings and knee-related PROMs. There was evidence of radiological knee OA in 40% of participants, and 47% showed signs of clinical OA. A history of previous severe knee injury and/or previous knee surgery was significantly correlated with the presence of radiological knee OA. There was no apparent correlation between the severity of radiological knee OA and PROMs. The presence of clinical knee OA was moderately correlated with impaired function.

### Perspective of the findings

The findings in this study support the findings of earlier research which identified that knee OA is prevalent amongst retired male professional footballers. The correlation between knee OA and previous severe knee injury and/or previous knee surgery is also consistent with previous studies.^[23]^ OA is not considered an occupational disease for professional footballers, but it has been shown to have a higher prevalence amongst retired male professional footballers when compared to those in the general population or in active footballers. It is reported that 18% of the general UK population sought treatment for knee OA and 8% have sought treatment for hip OA.^[16]^ The prevalence of knee OA (13%) amongst current professional footballers is significantly less than that amongst retired professional footballers.^[23]^

### Radiological versus clinical knee osteoarthritis

Radiological OA is not a reliable predictor of clinical outcomes in OA.^[24]^ In this pilot study, there was no significant correlation between radiological knee OA and PROMs. This is consistent with a broad body of literature highlighting that radiological findings are not necessarily consistent with clinical findings.^[25]^ Moderate correlation was found between clinical knee OA and KOOS-SP, indicating that pain and function may be a more useful guide to determine the presence and impact of knee OA in retired male professional footballers.

### Future directions: Clinical practice

This pilot study provides a reference for future studies to examine the complex interplay between imaging findings, clinical presentation, quality of life and other health outcomes amongst retired professional footballers with knee OA. Identifying those most at risk of negative sequelae may allow for more targeted interventions during and after a player’s career. These interventions may extend to workload monitoring, better surgical decision-making and lifestyle advice upon retirement. Recently, an After Career Consultation (ACC) was developed in order to empower the sustainable physical, mental and social health, and the quality of life of retired professional footballers.^[26]^ During the ACC, recently retired professional footballers receive evidence-based lifestyle advice which may mitigate their risk of developing OA.^[27]^ Further studies are required to assess the efficacy of any such interventions aimed at preventing or reducing the burden of knee OA amongst retired professional footballers.

### Future directions: Research

This pilot study provides an initial insight into the relationship between clinical knee OA, radiological OA and PROMs in a cohort of 15 retired male professional footballers. Expanding the study may provide additional insights which can be used to mitigate the risk of knee OA in retired male professional footballers.

A recent scoping review highlighted that there are very few studies examining the presence of musculoskeletal conditions in retired female professional footballers.^[28]^ Only two studies were identified which assessed the prevalence of knee OA in retired female professional footballers, prevalence ranging from 14% to 60%.^[28]^ Further studies are required to understand the impact of clinical knee OA and radiological knee OA on PROMs in this population.

### Limitations

Several methodological limitations should be acknowledged. Firstly, our study was only a pilot study conducted to help in the design of future larger cohort studies and defining support measures, such as the ACC.^[26]^ We therefore used only a small sample size, which might have affected the study’s external validity making it difficult to generalise the findings. However, our pilot study may pave the way for future studies investigating the relationship between clinical OA, radiological OA and PROMs. Using more advanced imaging (e.g. MRI) to assess for radiological OA may have provided more detailed findings.

Secondly, information about severe knee injuries and knee surgery was self-reported. Professional footballers can generally recall quite accurately the number of severe injuries and surgeries they had that led to at least four weeks without training or competition. However, recall bias cannot be categorically excluded.

Thirdly, it is very likely that knee injuries and knee surgeries have occurred prior to the development of knee OA. However, because of our cross-sectional design, such a time sequence is difficult to establish with certainty.

Lastly, our pilot study included key features, such as history of previous severe knee injury and knee surgery, career duration and level of play. Other details, such as career earnings and nationality, may have provided useful confounding data. Future studies may extend exclusion criteria to exclude any participants who have experienced trauma to the knee joint not directly related to playing football (e.g. motor vehicle accidents, injurious falls, etc.).

## Conclusion

Clinical knee OA correlates with PROMS amongst retired professional footballers but radiological OA does not. Further studies are required to understand the relationship between imaging findings, clinical presentation and PROMs amongst retired professional footballers with knee OA.

## Supplementary Information



## Figures and Tables

**Table 1:** Descriptive characteristics of retired professional footballers (N=15)

Age (years)	39 ± 3.6
Height (cm)	183 ± 5.0
Weight (kg)	82 ± 7.4
Body Mass Index (kg/m^2^)	24.6 ± 1.7
Career duration (years)	13 ± 2.8
Level of play (top league; %)	87
Educational level (%):	
No schooling completed	0
Nursery/Elementary school	0
High school	7
Vocational/technical school	7
College, university or equivalent	86
Retirement duration (years)	5.0 ± 4.1
Forced retirement (%)	13
Employed (%)	93

Data are expressed mean ± SD unless indicated otherwise.

**Table 2:** Knee-related injury, surgery, osteoarthritis and Patient-Reported Outcome Measures among retired professional footballers (N=15)

**Severe knee injury (%)**	
None	27
One or two	60
Three or more	13

**Knee surgery (%)**	
None	47
One or two	33
Three or more	20

**Radiological findings of knee osteoarthritis (%)**	
No radiological findings of osteoarthritis	7
Doubtful narrowing of joint space and possible osteophytic lipping	20
Definite osteophytes and possible narrowing of joint space	33
Moderate multiple osteophytes, definite narrowing of joint space, small pseudocystic areas with sclerotic walls and possible deformity of bone contour	13
Large osteophytes, marked narrowing of joint space, severe sclerosis and definite deformity of bone contour	27

**Radiological and clinical knee osteoarthritis (%)**	
No	73
Yes	27

**Radiological knee osteoarthritis (%)**	
No	60
Yes	40

**Clinical knee osteoarthritis (%)**	
No	53
Yes	47

***KOOS-SP***	88 ± 16

**Reference value KOOS-SP (mean)**	
General population	87–92
Young athletic population	86–99
Amateur footballers	87–96

**Global Physical Health** *	55 ± 7.1
**Global Mental Health** *	54 ± 9.9

Data are expressed mean ± SD unless indicated otherwise. KOOS-SP, Knee injury and Osteoarthritis Outcome Score Physical Function Short Form;

* mean score of 50 indicating the norm for the general population.
