# Supplementary material for: Osteoarthritic changes in the knees of recently retired male professional footballers: a pilot study
Source: S Afr J Sports Med. 2022 Jan 1;34(1):v34i1a12816. doi: 10.17159/2078-516X/2022/v34i1a12816 (PMC9924553; doi:10.17159/2078-516X/2022/v34i1a12816)
Supplement: Supplementary file 1 [file 2078-516X-34-v34i1a12816-s001.pdf]

Osteoarthritic changes in the knees of recently retired male professional footballers: a pilot study

Supplementary Data

1. Consent

2. Characteristics

2.1 What is your age (in years)?
2.2 What is your gender?
2.3 How tall are you (in cm or ft)?
2.4 How much do you weigh (in kg)?
2.5 For how many years were you a professional footballer?
2.6 At which level did you (principally) play?
2.7 For how many years have you been retired from professional football?
2.8 Did you quit professional football voluntarily?
2.9 What is the highest level of education you have completed?
2.10 Do you have currently a paid job?

3. Injuries and surgeries

3.1 How many severe knee injuries have you had during your professional football career?
3.2 How many knee surgeries have you had during your professional football career?
3.3 Have you been diagnosed with knee osteoarthritis?

4. Knee functions

4.1 During the last week, how severe was your knee stiffness after first wakening in the morning?
4.2 What amount of knee pain have you experienced the last week during twisting/pivoting on your knee?
4.3 What amount of knee pain have you experienced the last week during straightening knee fully?
4.4 What amount of knee pain have you experienced the last week during going up or down stairs?
4.5 What amount of knee pain have you experienced the last week during standing upright?
4.6 For rising from sitting, indicate the degree of difficulty you have experienced in the last week due to your knee.
4.7 For bending to floor/pick up an object, indicate the degree of difficulty you have experienced in the last week due to your knee.

5. Quality of life

5.1 In general, would you say your health is:
5.2 In general, would you say your quality of life is:
5.3 In general, how would you rate your physical health?
5.4 In general, how would you rate your mental health, including your mood and your ability to think?
5.5 In general, how would you rate your satisfaction with your social activities and relationships?
5.6 In general, please rate how well you carry out your usual social activities and roles. (This includes activities at home, at work and in your community, and responsibilities as a parent, child, spouse, employee, friend, etc.) ......
5.7 To what extent are you able to carry out your everyday physical activities such as walking, climbing stairs, carrying groceries, or moving a chair?
5.8 In the past 7 days, how often have you been bothered by emotional problems such as feeling anxious, depressed or irritable?
5.9 In the past 7 days, how would you rate your fatigue on average?
5.10 In the past 7 days, how would you rate your pain on average?
